# Delayed response to maintenance therapy after first-line chemotherapy in metastatic intrahepatic cholangiocarcinoma: a case report

**DOI:** 10.1186/s13256-017-1443-8

**Published:** 2017-09-26

**Authors:** Roberta Marciano, Alberto Servetto, Cataldo Bianco, Roberto Bianco

**Affiliations:** 1 Department of Clinical Medicine and Surgery, Oncology Division, University of Naples Federico II, Naples, Italy; 2 Department of Experimental and Clinical Medicine, University of Catanzaro Magna Grecia, Catanzaro, Italy

**Keywords:** Intrahepatic cholangiocarcinoma, Maintenance chemotherapy, Gemcitabine

## Abstract

**Background:**

Intrahepatic cholangiocarcinoma is an aggressive tumor originating in the epithelium of the bile duct, often associated with distant dissemination. The prognosis is poor and treatment is challenging due to low response rate to standard chemotherapy and lack of targeted therapies.

**Case presentation:**

Here we report the case of a 74-year-old white woman affected by intrahepatic cholangiocarcinoma with metastatic involvement of spleen, lung, peritoneum, and intra-abdominal lymph nodes. As first-line chemotherapy, she was given cisplatin-gemcitabine chemotherapy. The treatment was well tolerated with the exception of grade 1 constipation and a single episode of grade 4 thrombocytopenia occurring after the fourth course. After the first three courses of chemotherapy a computed tomography scan evaluation demonstrated no change; her CA19-9 levels were slightly decreased. However, after the sixth course of chemotherapy a computed tomography scan revealed a dimensional enlargement of the lung metastases; her CA19-9 levels increased. She was then treated with gemcitabine alone. After 2 months of gemcitabine monotherapy a significant regression of lung and spleen metastases, as well a CA19-9 level reduction, occurred. Eight months after the start of gemcitabine monotherapy no signs of progression were reported.

**Conclusions:**

Treatment of metastatic intrahepatic cholangiocarcinoma with gemcitabine as maintenance therapy after first-line chemotherapy could be continued until clear evidence of disease progression since delayed responses are possible.

## Background

Intrahepatic cholangiocarcinomas (ICCs) comprise all tumors originating in the bile duct epithelium within the liver and are the second most common primary liver malignancy. ICC is a relatively rare malignancy with an age-adjusted incidence rate between 0.4/100,000 and 1.8/100,000 per year throughout Europe [1]. The overall survival (OS) is approximately 6 months from the time of diagnosis, since the majority of patients (90%) are not eligible for curative resection. The main risk factors for ICC are primary sclerosing cholangitis, human immunodeficiency virus (HIV) infection, diabetes, tobacco smoking, cirrhosis, and hepatitis C infection [2–5]. For patients with unresectable disease but in the absence of systemic dissemination, palliative chemoradiotherapy or palliative ablation and chemoembolization are major options. For metastatic disease chemotherapy is increasingly being applied. The gemcitabine/cisplatin combination therapy is currently the best option for metastatic ICC [6]. The alternatives are represented by fluoropyrimidine-based or other gemcitabine-based chemotherapy regimens. Here we report the case of a patient with metastatic ICC which showed a delayed and durable response to continuation maintenance therapy with gemcitabine.

## Case presentation

**Fig. 1:**
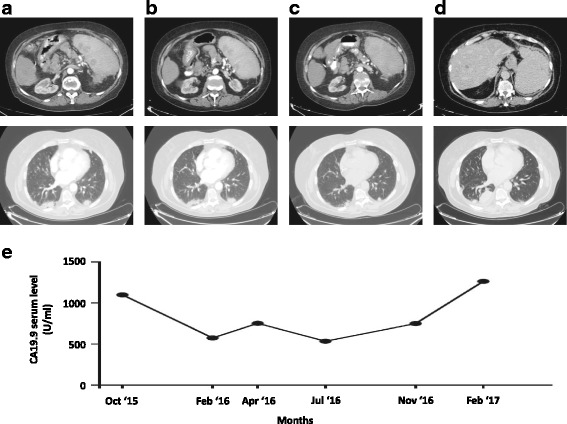
Computed tomography scans and CA19-9 serum level evaluation during treatment. **a** Basal computed tomography scan evaluation; spleen and lung lesions are evident. **b** Computed tomography evaluation after six courses of cisplatin-gemcitabine; no significant changes in lung and spleen lesion were observed. **c** Computed tomography evaluation after 4 months of gemcitabine maintenance therapy revealed a complete remission of spleen lesions and a significant regression of lung metastases. **d** Computed tomography evaluation showing disease progression after 10 months of maintenance therapy. **e** CA19-9 serum levels through time since first diagnosis

In September 2015 a 74-year-old white woman presented with abdominal pain, pyrosis, and weight loss. Her medical history included cholecystectomy for cholelithiasis in 2010. Her blood analyses were normal. A physical examination was unremarkable. A computed tomography (CT) scan showed an intrahepatic neoplastic lesion of 38 mm localized in the gallbladder bed associated with multiple spleen, pulmonary, and peritoneal metastases. Serum levels of CA19-9 were increased (1100 U/mL; reference range less than 37 U/mL). She was then referred to our oncology division. The Eastern Cooperative Oncology Group (ECOG) performance status (PS) was equal to 0; a physical examination found only mild abdominal tenderness. In October 2015 an ultrasound-guided biopsy was performed on the intrahepatic lesion. Pathological analysis revealed the presence of bile duct adenocarcinoma cells; immunohistochemistry showed strong expression for CK19. A pulmonary biopsy on one of the secondary lesions confirmed the diagnosis of lung metastasis. From November 2015 to January 2016 she underwent first-line chemotherapy with cisplatin (25 mg per square meter of body surface area) followed by gemcitabine (1000 mg per square meter), each administered on days 1 and 8 every 3 weeks [6]. After three courses of treatment, a CT scan showed a condition of stable disease assessed by Response Evaluation Criteria in Solid Tumors (RECIST) evaluation criteria. Her serum levels of CA19-9 were slightly decreased to 576 U/mL. No signs of bone marrow toxicity were observed; grade 1 of gastrointestinal toxicity was recorded (nausea and vomiting). She was then treated with an additional three courses of cisplatin-gemcitabine. A following CT scan revealed a dimensional increase of lung metastases with a 10% increase by RECIST criteria, whereas no changes were observed in the other lesions; her CA19-9 serum levels were increased to 753 U/mL. Grade 4 thrombocytopenia was observed soon after the fourth cycle of chemotherapy; the doses were then reduced by 10%. Grade 1 constipation and nausea were reported for the remaining courses. Her PS had not deteriorated; her liver and renal functions were normal. We then decided to treat her with gemcitabine alone as maintenance therapy starting in April 2016, with the following schedule: 1000 mg per square meter, administered on days 1, 8, and 15 every 4 weeks. After the second cycle, grade 3 thrombocytopenia and lower limb lymphedema were observed and the doses were reduced by 20%. In August 2016 a CT scan revealed a significant dimensional regression of lung lesions (50% reduction as assessed by RECIST evaluation criteria) and a complete regression of spleen lesions (Fig. 1 a–c); her CA19-9 serum levels were decreased to 536 U/mL. She continued maintenance therapy with gemcitabine alone for 10 months until February 2017 when a CT scan showed lung disease progression and new liver lesions (Fig. 1 d). At the same time, her CA19.9 serum level increased to 1265 U/mL (Fig. 1 e).

## Discussion

While an accepted modality in other cancer types, such as lung cancer, maintenance therapy in gastrointestinal malignancies is not routinely used. In the setting of metastatic colorectal cancer, maintenance therapy has been recently evaluated in phase III randomized trials. For example, combination therapy incorporating capecitabine and bevacizumab, as maintenance treatment after induction therapy with capecitabine, oxaliplatin, and bevacizumab (CAPOX-B), significantly improved progression-free survival (PFS) with excellent tolerability, although the incidence of hand-foot syndrome was increased [7]. Maintenance capecitabine monotherapy has also been evaluated after induction therapy with oxaliplatin and infusional 5-fluorouracil (FOLFOX-4) in patients with metastatic colorectal cancer with very low incidence of side effects [8]. Little data are available for metastatic pancreatic cancer and no data for bile duct cancers. Maintenance therapy with gemcitabine after chemoradiation has been proposed for patients with locally advanced pancreatic cancer and, despite the limitation of a retrospective analysis, an increase in OS is suggested [9]. Reure and colleagues reported a retrospective series of patients with metastatic pancreatic cancer treated with maintenance capecitabine without signs of progression after first-line leucovorin, fluorouracil, irinotecan, and oxaliplatin (FOLFIRINOX) chemotherapy with a median OS of 17 months and a median PFS of 5 months [10]. Doherty *et al*. have recently reported retrospective data suggesting a survival benefit for patients affected by biliary tract cancer receiving nine or more cycles of chemotherapy, with manageable toxicity [11]. Here we describe the case of a women affected by metastatic ICC treated with first-line cisplatin-gemcitabine chemotherapy. In the phase III randomized trial by Valle and colleagues, the combination of cisplatin-gemcitabine was compared to gemcitabine in patients with locally advanced or metastatic biliary tract cancer [6]. The cisplatin-gemcitabine group achieved a significantly better tumor control rate (81.4 versus 71.8%, *p* = 0.049), an increased median PFS (8.0 months versus 5.0 months, *p* < 0.001), and an improved median OS (11.7 months versus 8.1 months, *p* < 0.001) with more incidence of bone marrow toxicity (mainly neutropenia in the combination arm). In our case, our patient achieved disease stabilization as best overall response after the first three courses of chemotherapy, with a dimensional increase in lung metastases after the sixth course, although it was not sufficient to qualify for progression. Maintenance therapy with gemcitabine was well tolerated and, surprisingly, resulted in a significant clinical delayed response, with a dimensional regression of both lung and spleen metastases. Usually, traditional chemotherapy is associated with rapid but transient rather than delayed durable response, the latter being frequently observed for novel immunotherapy drugs. It could be hypothesized that, in our case, the chemotherapy treatment had unmasked tumor antigens and the antigen release induced an immune-mediated antitumor response, without any significant change in white blood cell (WBC), neutrophil, monocyte, and lymphocytes counts. Recent data suggest that the anticancer effect of chemotherapy is also due to the interaction with innate and adaptive immune system, with various complex mechanisms [12]. This new evidence represents the rationale to design clinical trials testing the combination of cytotoxic agents and immune checkpoint inhibitors.

## Conclusions

Our case suggests that treatment of metastatic ICC with first-line chemotherapy could be continued until clear evidence of disease progression since delayed responses are possible. With this in mind, gemcitabine monotherapy appears to be a reasonable option if well tolerated and a good performance status (PS) is maintained through the treatment.

## Abbreviations

CT: Computed tomography
ICC: Intrahepatic cholangiocarcinoma
OS: Overall survival
PFS: Progression-free survival
PS: Performance status
RECIST: Response Evaluation Criteria in Solid Tumors
